# Supplementary material for: Molecular insights into Vibrio cholerae’s intra-amoebal host-pathogen interactions
Source: Nat Commun. 2018 Aug 27;9:3460. doi: 10.1038/s41467-018-05976-x (PMC6110790; doi:10.1038/s41467-018-05976-x)
Supplement: Supplementary file 1 — Description of Additional Supplementary Files [file 41467_2018_5976_MOESM1_ESM.pdf]

Description of Additional Supplementary Files

File Name: Supplementary Movie 1
Description: 3D reconstruction of a *V. cholerae*-colonized amoebal contractile vacuole.

File Name: Supplementary Movie 2
Description: Intra-vacuolar dynamics of WT *V. cholerae*.

File Name: Supplementary Movie 3
Description: Intra-vacuolar dynamics of ΔflaA *V. cholerae*.

File Name: Supplementary Movie 4
Description: Intra-vacuolar dynamics of ΔpomB *V. cholerae*.

File Name: Supplementary Movie 5
Description: Time-lapse confocal microscopy movie of a colonized contractile vacuole co-housing WT (dsRed-tagged) and ΔflaA (GFP-tagged) bacteria. See snapshots in Fig. 6c.

File Name: Supplementary Movie 6
Description: Short section of Supplementary Movie 5 at a slower speed showing the rupture of the contractile vacuole.

File Name: Supplementary Movie 7
Description: Short section of Supplementary Movie 5 at a slower speed showing the cyst lysis and the spread.
