# Supplementary material for: Molecular insights into Vibrio cholerae’s intra-amoebal host-pathogen interactions
Source: Nat Commun. 2018 Aug 27;9:3460. doi: 10.1038/s41467-018-05976-x (PMC6110790; doi:10.1038/s41467-018-05976-x)
Supplement: Supplementary file 10 — Supplementary Information [file 41467_2018_5976_MOESM10_ESM.pdf]

Molecular insights into *Vibrio cholerae*'s intra-amoebal host-pathogen interactions

Van der Henst *et al*.

Supplementary Information

Supplementary Figures

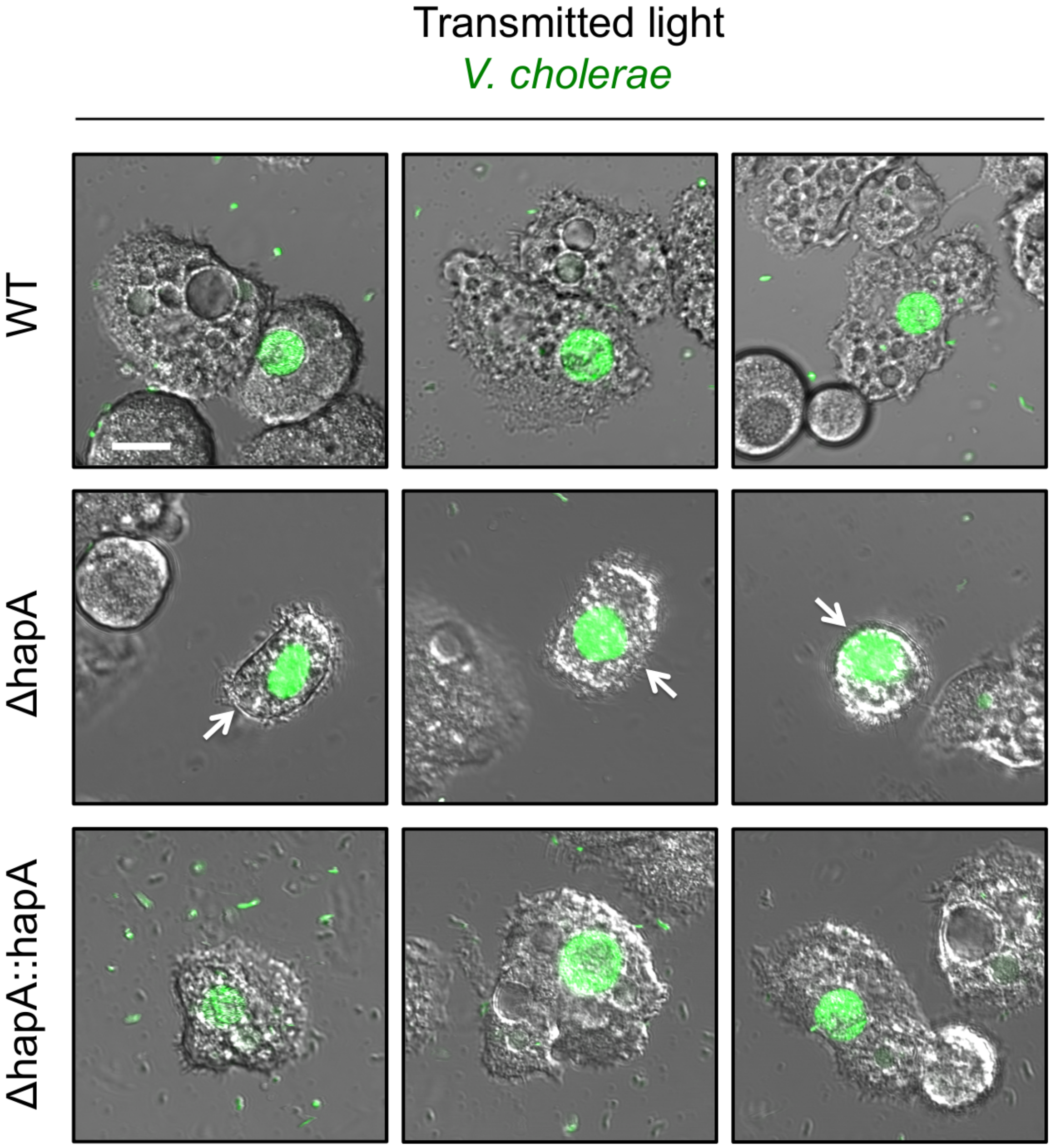

Supplementary Figure 1. Aberrant amoebal morphology caused by hemagglutinin protease (HapA)-deficient *V. cholerae*. Additional confocal images of GFP-tagged bacteria and amoebae as in Fig. 2a. Shown are merged images of the transmitted light channel and the green channel. Bacterial strains depicted: WT, ΔhapA, and the complemented mutant (ΔhapA::hapA). White arrows depict aberrant morphotypes.

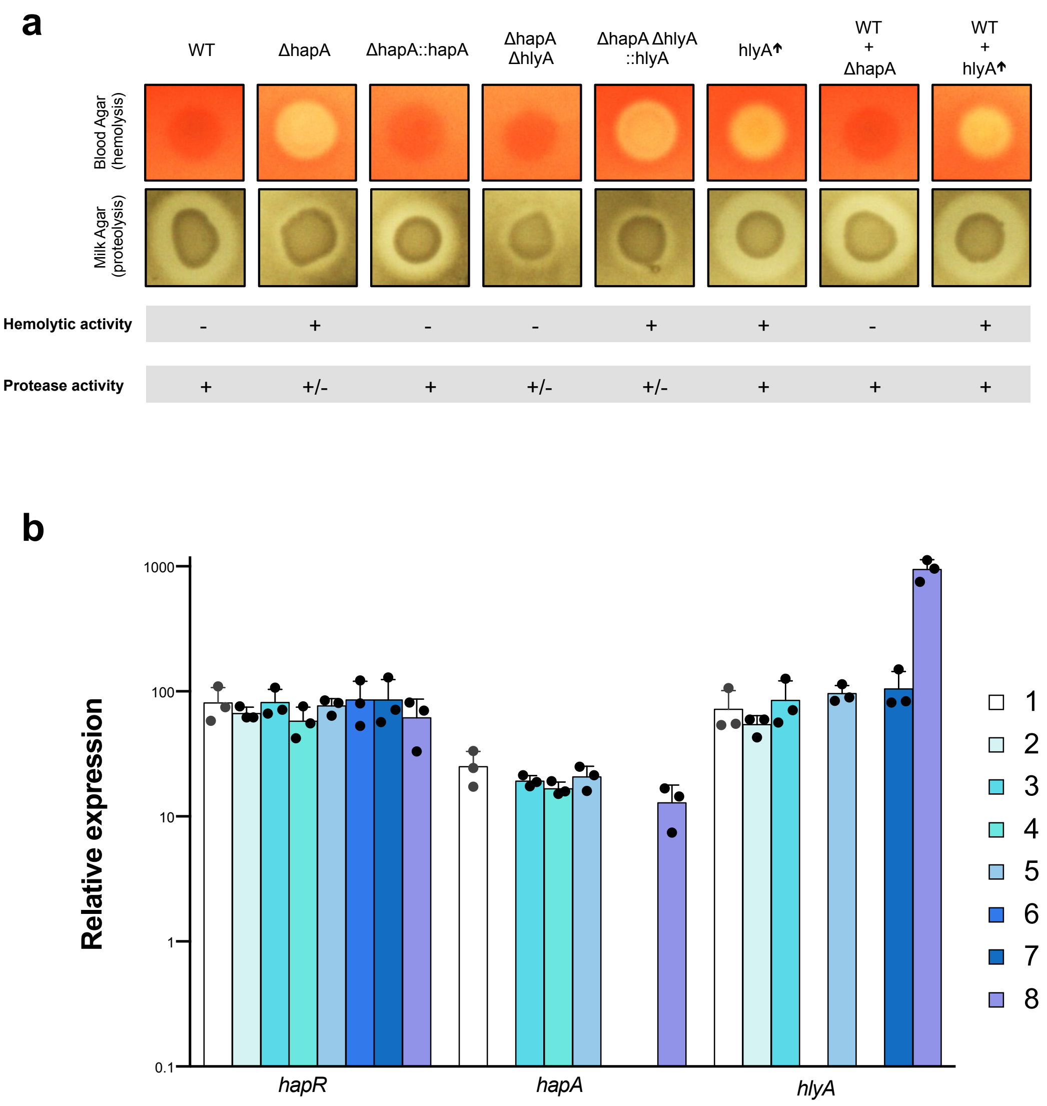

Supplementary Figure 2. Characterization of genetically engineered *V. cholerae* strains. Diverse *V. cholerae* strains were tested *in vitro* **(a)** for hemolytic activity on blood agar plates and for protease activity on milk agar plates or **(b)** for expression of representative genes by qRT-PCR. The strains tested in panel (a) are WT, ΔhapA, ΔhapA-complemented (ΔhapA::hapA), the protease and hemolysin double mutant without *hlyA* complementation (ΔhapAΔhlyA) and with *hlyA* complementation (ΔhapAΔhlyA::hlyA), and a *hlyA*-overexpression strain (hlyA↑). The last two rows show 1:1 mixtures of both indicated strains. **(b)** Relative gene expression confirming proficient complementation or overexpression of *hapA* and *hlyA*. Transcript levels were scored by qRT-PCR and normalized to *gyrA* (shown as relative expression values on the *Y*-axis). The gene encoding the quorum-sensing master regulator HapR (*hapR*) serves as negative control (no difference in its expression in any of the tested strains). Strains tested: 1, WT; 2, ΔhapA; 3, ΔhapA::hapA; 4, ΔhlyA; 5, ΔhlyA::hlyA; 6, ΔhapAΔhlyA; 7, ΔhapAΔhlyA::hlyA; 8, hlyA↑. Data are averages from three independent biological replicates (± s.d.).

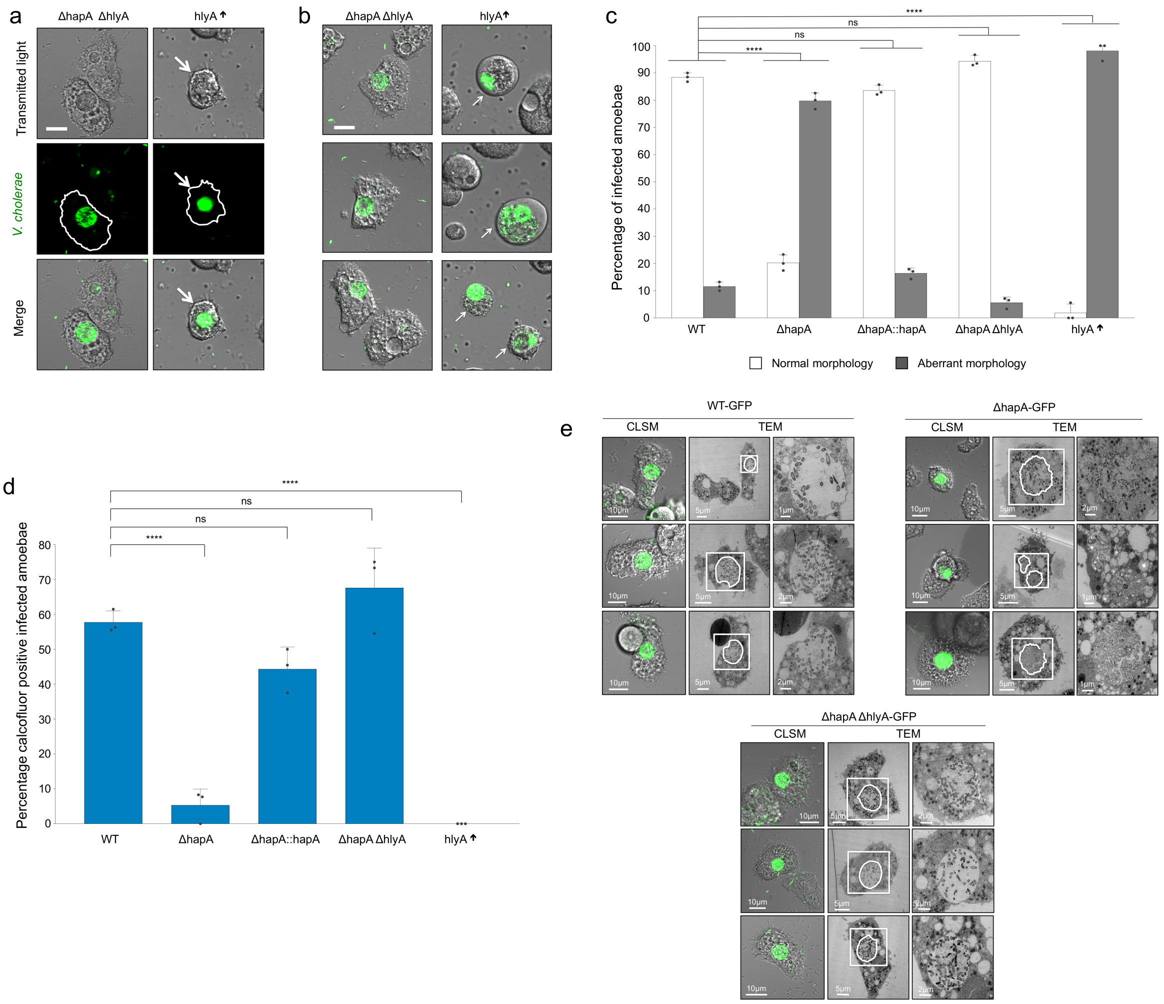

Supplementary Figure 3. The hemolysin of *V. cholerae* intoxicates *A. castellanii.* (a), **(b)** Additional confocal micrographs of amoebae infected with GFP-tagged ΔhapAΔhlyA and hlyA↑ strains of *V. cholerae.* Details as in Fig. 3a. Shown are in panel (a) the transmitted light channel, the green channel, and a merged image of both channels and in panel (b) merged channel images only. Scale bars for (a) and (b): 10 µm. The white arrows show aberrant morphotypes. **(c), (d)** Quantification of normal or aberrant morphologies and deposition of cellulose by amoebae infected with hemolysin-overactive *V. cholerae* strains. Details as in Fig. 2b and e. Values for WT, ΔhapA, and ΔhapA::hapA are the same as in Fig. 2 to compare the values to the ΔhapAΔhlyA and hlyA↑ strains. Statistics are based on a one-way ANOVA with ****, $p \leq 0.0001$; ns, $p > 0.05$. **(e)** Malformation of the contractile vacuole in amoebae infected by protease-deficient and, therefore, hemolysin-overactive *V. cholerae* strains. Additional CLEM images as in Fig. 3c show the malformation of the contractile vacuole in *hapA*-deficient strains (ΔhapA) compared to WT or *hapA*- and *hlyA*-deficient strains (ΔhapAΔhlyA).

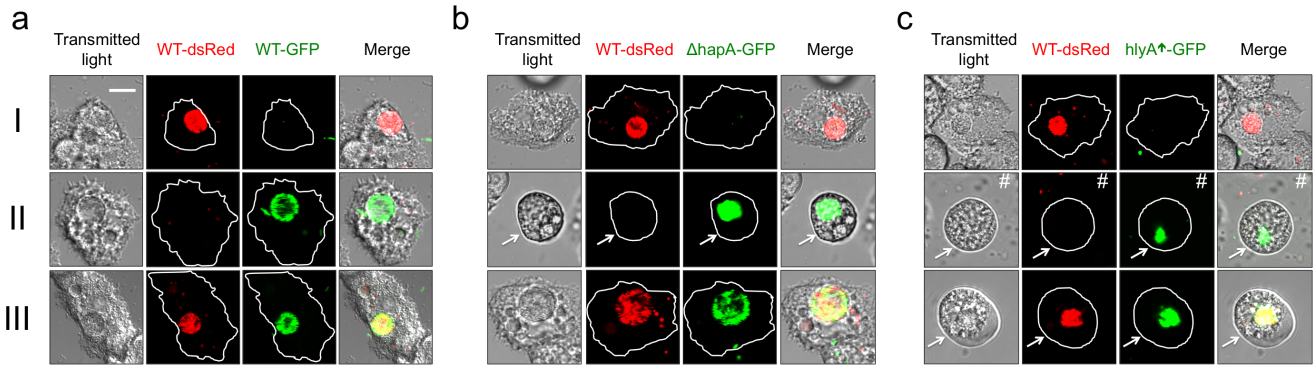

Supplementary Figure 4. Intra-vacuolar activities of HapA and HlyA. Amoebae infected by a 1:1 mixture of the two strains were imaged by confocal microscopy at 20 hours p.p.c. The WT is tagged with dsRed in all images, while the GFP-tagged strain corresponds to **(a)** a second WT, **(b)** ΔhapA, **(c)** and hlyA↑. The micrographs depict amoebae colonized (I) by the dsRed-tagged strain, (II) the GFP-tagged strain, (III) or both strains. For each lane, the transmitted light channel, the red channel, the green channel, and a merged image of all three channels are displayed. Quantifications of these experiments from three independent biological replicates are given in Supplementary Table 2. #Micrograph shows a single event observed out of 6,000 amoebae counted.

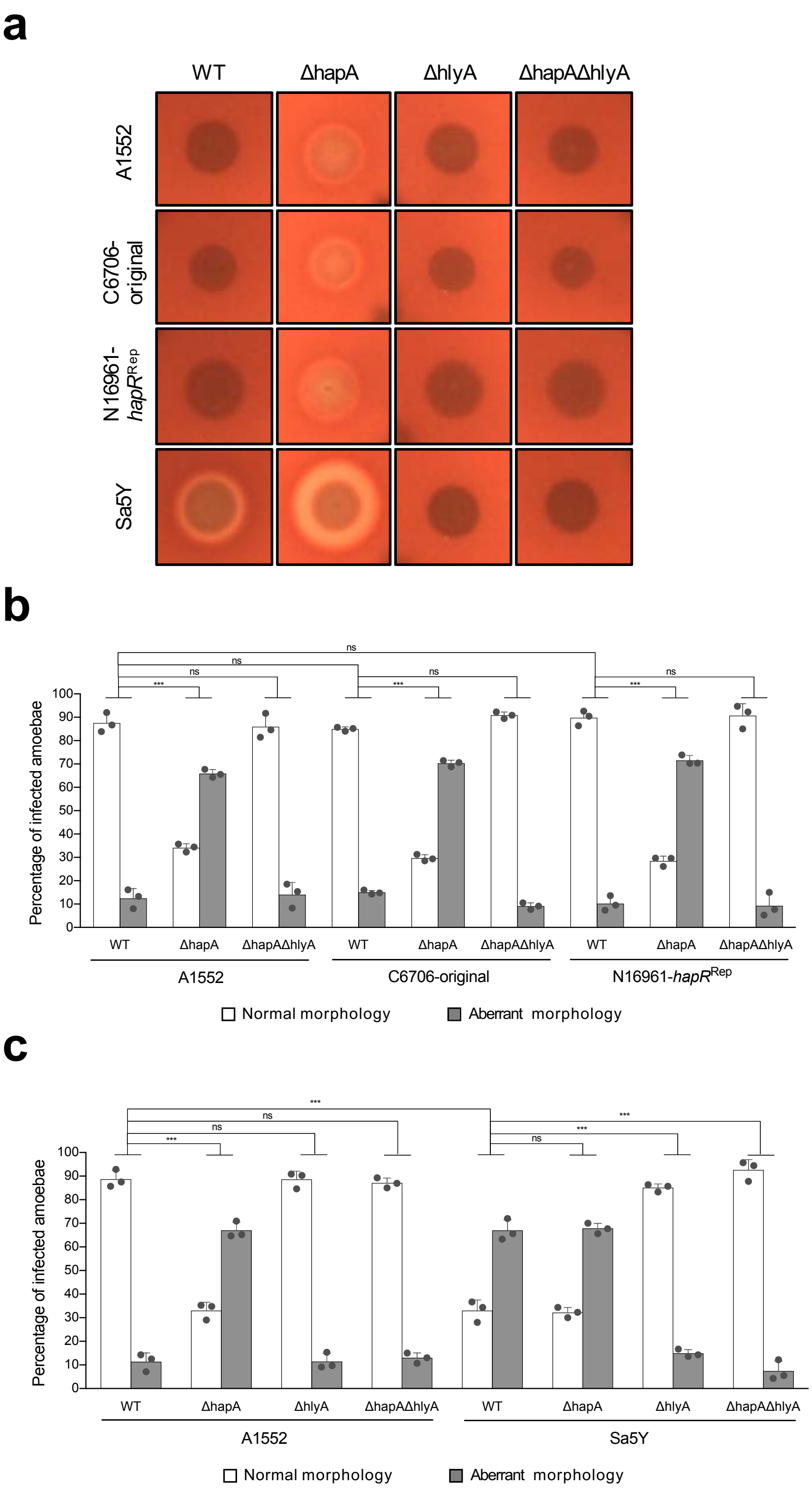

Supplementary Figure 5. Hemolysin-based intoxication of *V. cholerae* strains is common in pandemic and environmental *V. cholerae* strains. (a) Pandemic *V. cholerae* strains A1552, C6706-original (quorum sensing-proficient old stock of C6706), N16961-*hap*^{Rep} (quorum sensing-repaired variant of reference strain N16961), and environmental isolate Sa5Y were tested for hemolytic activity on blood agar plates. **(b, c)** Quantification of normal and aberrant morphologies of amoebae infected with mCherry-tagged pandemic (b) or environmental (c) *V. cholerae* strains as mentioned in panel (a) at 20 hours p.p.c. For each strain, the WT and its ΔhapA and ΔhapAΔhlyA variant was tested. For (c) the ΔhlyA single mutant was also tested. Values represent averages from three independent experiments (± s.d.). Statistics are based on a one-way ANOVA with ***, $p \leq 0.001$; ns, $p > 0.05$.

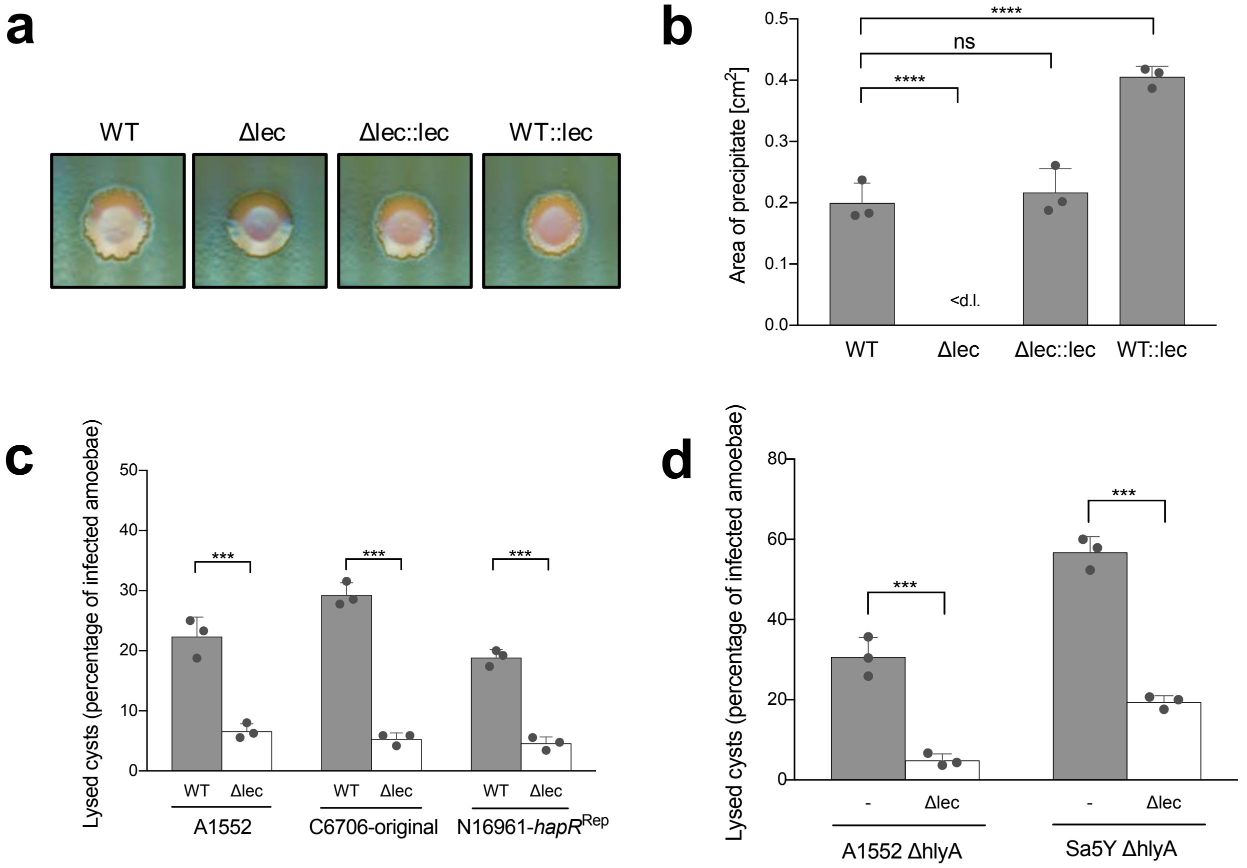

Supplementary Figure 6. Contribution of lecithinase activity to cyst lysis in diverse *V. cholerae* strains. (a) Quantification of *in vitro* lecithinase activity. *V. cholerae* strains were tested for lecithinase activity on egg yolk agar plates. Lecithinase degrades lecithin that is present in egg yolk, thereby producing an insoluble and opaque precipitate around the colonies. **(b)** Quantification of the area of precipitation. Bacterial strains tested were WT, Δlec, Δlec-complemented (Δlec::lec), and the *lec*-merodiploid strain (WT::lec). The graph shows the averages of three independent biological experiments (± s.d.). **(c)** Quantification of lysed cysts that were infected by mCherry-tagged *V. cholerae* A1552, C6706-original and N16961-*hapR*^{Rep} wild-type strain (WT) and lecithinase-minus (Δlec) derivatives thereof at 30 hours p.p.c. (as in Fig. 5b). **(d)** Same as panel (c), except that the amoebae were infected with the environmental isolate Sa5Y and scored for proficient cyst lysis in the presence or absence of *lec*. All strains were *hlyA*-negative to avoid premature intoxication by strain Sa5Y. The pandemic strain A1552 served as control. The graphs in panels (c) and (d) represent average values from three independent biological replicates. Statistics are based on one-way ANOVA. ****, $p \leq 0.0001$; ***, $p \leq 0.001$; **, $p \leq 0.01$; ns, $p > 0.05$.

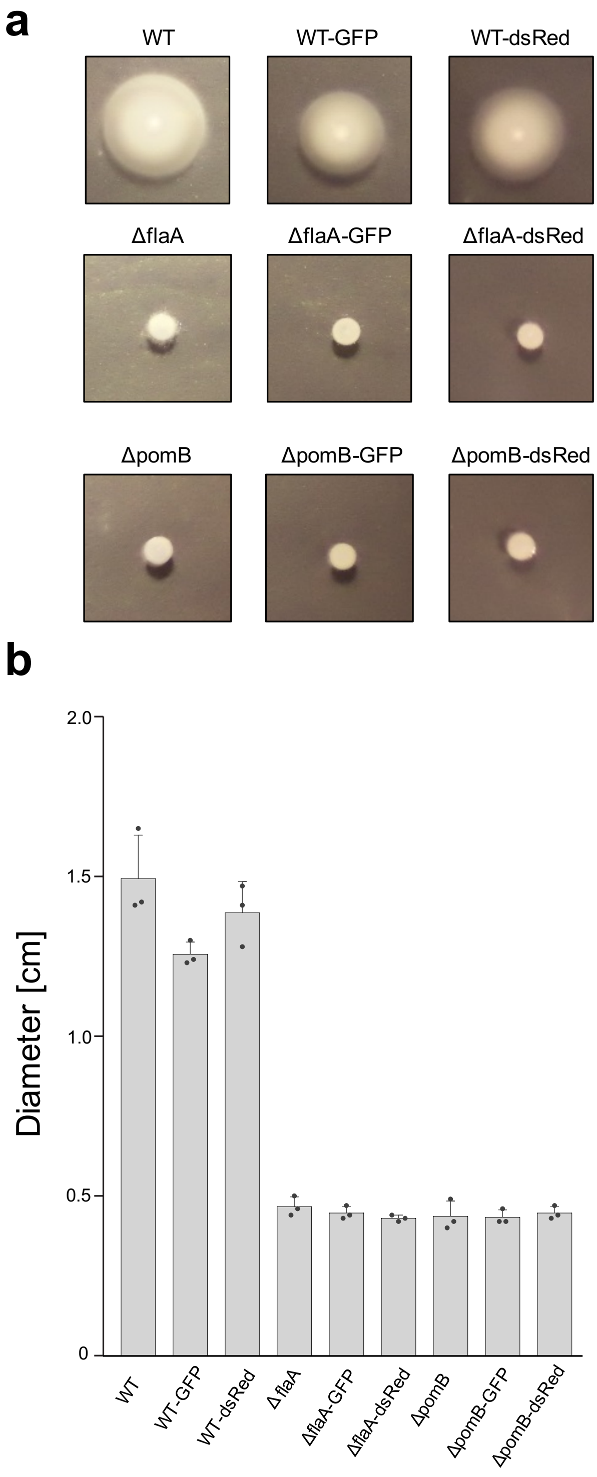

Supplementary Figure 7. Confirmation of *in vitro* motility of genetically engineered *V. cholerae* strains. The *V. cholerae* WT, ΔflaA, and ΔpomB strains (without or with constitutively expressed *gfp* or *dsRed*, as indicated) were tested for flagellum-based motility on soft agar plates. **(a)** Representative images. **(b)** Data from three independent biological replicates were quantified based on the swarming diameter (± s.d.).

Supplementary Tables

Supplementary Table 1. Bacterial strains and plasmids used in this study.

Strains and plasmids	Genotype (*) / Description (#)	Internal strain number	Reference
V. cholerae strains			
A1552 (WT)	Wild-type, O1 El Tor Inaba, Rif^R	MB_1	(Yildiz and Schoolnik, 1998)[1]
A1552-GFP	A1552 with mTn7-*gfp*; RifR, Cm^R, $Gent^R$	MB_459	(Blokesch, 2012a)[2]
A1552-dsRed	A1552 with mTn7-*dsRed.T3[DNT]*; Rif^R, $Gent^R$	MB_1524	(Borgeaud *et al.*, 2015)[3]
A1552-mCherry	A1552 with mTn7-*mCherry*; Rif^R, $Gent^R$	MB_5536	This study
A1552ΔhapA	A1552 deleted for *hapA* (VCA0865; TransFLP); Rif^R	MB_3991	This study
A1552ΔhapA-GFP	ΔhapA with mTn7-*gfp*; Rif^R, Cm^R, $Gent^R$	MB_4080	This study
A1552ΔhapA::hapA	ΔhapA complemented with *hapA* on chromosome; Rif^R	MB_5067	This study
A1552ΔhapA::hapA-GFP	ΔhapA::hapA with mTn7-*gfp*; Rif^R, Cm^R, $Gent^R$	MB_5111	This study
A1552ΔhapA-mCherry	A1552 with *cat* insertion in *hapA* (VCA0865) and mTn7-*mCherry*; Rif^R, $Gent^R$, Cm^R	MB_6098	This study
A1552ΔhlyA	A1552 deleted for *hlyA* (VCA0219; TransFLP); Rif^R	MB_3935	(Metzger *et al.*, 2016)[4]
A1552ΔhlyA-GFP	A1552 deleted for *hlyA* (VCA0219, TransFLP) with mTn7-*gfp*; Rif^R, Cm^R, $Gent^R$	MB_6065	This study
A1552ΔhlyA::hlyA	ΔhlyA complemented with *hlyA* on chromosome*;* Rif^R	MB_4939	This study
A1552ΔhapAΔhlyA	A1552 deleted for *hapA* (VCA0865; TransFLP) and *hlyA* (VCA0219; TransFLP); Rif^R	MB_4199	This study
A1552ΔhapAΔhlyA-GFP	ΔhapAΔhlyA with mTn7-*gfp*; Rif^R, Cm^R, $Gent^R$	MB_5615	This study
A1552ΔhapAΔhlyA-dsRed	ΔhapAΔhlyA with mTn7-*dsRed.T3[DNT]*; Rif^R, $Gent^R$	MB_5615	This study
A1552ΔhapAΔhlyA::hlyA	ΔhapAΔhlyA complemented with *hlyA* on chromosome*;* Rif^R	MB_4991	This study
A1552ΔhlyA-mCherry	A1552 with *aph* insertion in *hlyA* (VCA0219) and mTn7-*mCherry*; Rif^R, $Gent^R$, Kan^R	MB_6095	This study
A1552ΔhapAΔhlyA-mCherry	A1552 with *cat* insertion in *hapA*, *aph* insertion in *hlyA* and mTn7-*mCherry*; Rif^R, $Gent^R$, Cm^R, Kan^R	MB_6101	This study
ΔlacZ::pheS*	A1552 with construct ΔlacZ::FRT-Kan-pheS*-FRT; Rif^R, $cPhe^S$	MB_4609	This study
hlyA↑ (A1552-hlyA↑)	ΔlacZ::pheS* transformed with construct containing *hlyA* preceded by strong $P_{A1/04/03}$ promoter (ΔlacZ::hlyA; Trans2); Rif^R, $cPhe^R$	MB_4979	This study
hlyA↑-GFP (A1552-hlyA↑-GFP)	hlyA↑ with mTn7-*gfp*; Rif^R, Cm^R, $Gent^R$	MB_5060	This study
A1552ΔflaA	A1552 deleted for *flaA* (VC2188)*;* Rif^R	MB_1996	This study
A1552ΔflaA-GFP	ΔflaA with mTn7-*gfp*; Rif^R, Cm^R, $Gent^R$	MB_5434	This study
A1552ΔflaA-dsRed	ΔflaA with mTn7-*dsRed.T3[DNT]*; Rif^R, $Gent^R$	MB_5435	This study
A1552ΔpomB	A1552 deleted for *pomB* (VC0893; TransFLP), Rif^R	MB_4641	This study
A1552ΔpomB-GFP	ΔpomB with mTn7-*gfp*; Rif^R, Cm^R, $Gent^R$	MB_4797	This study
A1552ΔpomB-dsRed	ΔpomB with mTn7-*dsRed.T3[DNT]*; Rif^R, $Gent^R$	MB_4798	This study
A1552Δlec	A1552 deleted for *lec* (VCA0218 or *tlh*; TransFLP); Rif^R	MB_4189	(Metzger *et al.*, 2016)[4]
A1552Δlec::lec	Δlec complemented with *lec* on chromosome*;* Rif^R	MB_4841	This study

WT::lec (A1552::lec)	A1552 with additional copy of *lec;* Rif^R	MB_4840	This study
A1552Δlec-GFP	Δlec with mTn7-*gfp*; Rif^R, Cm^R, $Gent^R$	MB_5879	This study
A1552Δlec::lec-GFP	Δlec::lec with mTn7-*gfp*; Rif^R, Cm^R, $Gent^R$	MB_5881	This study
WT::lec-GFP (A1552::lec-GFP)	WT::lec with mTn7-*gfp*; Rif^R, Cm^R, $Gent^R$	MB_5880	This study
A1552Δlec-mCherry	A1552 with *aph* insertion in *lec* (VCA0218 or *tlh*) and mTn7-*mCherry*; Rif^R, $Gent^R$, Kan^R	MB_6104	This study
A1552ΔlecΔhlyA-mCherry	A1552 with *cat* insertion in *lec, aph* insertion in *hlyA* and mTn7-*mCherry*; Rif^R, $Gent^R$, Cm^R, Kan^R	MB_6132	This study
C6706-original	C6706-original; O1 El Tor Inaba; isolated in 1991, Peru; $Strep^S$ (original isolate before introduction of Streptomycin resistance-causing mutation; non-mutated *luxO*).	MB_4522	Gift from J. Mekalanos (Harvard)
C6706-original-mCherry	C6706-original with mTn7-*mCherry*; $Gent^R$	MB_6056	This study
C6706-originalΔhlyA-mCherry	C6706-original with *aph* insertion in *hlyA* (VCA0219) and mTn7-*mCherry*; $Gent^R$, Kan^R	MB_6096	This study
C6706-originalΔhapA-mCherry	C6706-original with *cat* insertion in *hapA* (VCA0865) and mTn7-*mCherry*; $Gent^R$, Cm^R	MB_6099	This study
C6706-originalΔhapA ΔhlyA-mCherry	C6706-original with *cat* insertion in *hapA*, *aph* insertion in *hlyA* and mTn7-*mCherry*; $Gent^R$, Cm^R, Kan^R	MB_6102	This study
C6706-originalΔlec-mCherry	C6706-original with *aph* insertion in *lec* (VCA0218 or *tlh*) and mTn7-*mCherry*; $Gent^R$, KanR	MB_6105	This study
N16961-*hapR*^{Rep}	N16961 with repaired *hapR* frameshift mutation (based on suicide plasmid-mediated allelic exchange); $Strep^R$,	MB_5663	This study
N16961-*hapR*^{Rep}-mCherry	N16961-*hapR*^{Rep} with mTn7-*mCherry*; $Strep^R$, $Gent^R$	MB_6057	This study
N16961-hapR^{Rep}ΔhlyA-mCherry	N16961-*hapR*^{Rep} with *aph* insertion in *hlyA* (VCA0219) and mTn7-*mCherry*; $Strep^R$, $Gent^R$, Kan^R	MB_6097	This study
N16961-*hapR*^{Rep}ΔhapA-mCherry	N16961-*hapR*^{Rep} with *cat* insertion in *hapA* (VCA0865) and mTn7-*mCherry*; $Strep^R$, $Gent^R$, Cm^R	MB_6100	This study
N16961-*hapR*^{Rep} ΔhapAΔhlyA-mCherry	N16961-*hapR*^{Rep} with *cat* insertion in *hapA*, *aph* insertion in *hlyA* and mTn7-*mCherry*; $Strep^R$, $Gent^R$, Cm^R, Kan^R	MB_6103	This study
N16961-*hapR*^{Rep}Δlec-mCherry	N16961-*hapR*^{Rep} with *aph* insertion in *lec* (VCA0218 or *tlh*) and mTn7-*mCherry*; $Strep^R$, $Gent^R$, Kan^R	MB_6106	This study
Sa5Y	non-O1/O139 environmental *V. cholerae* strains; isolated from the central California coast	MB_353	(Keymer *et al.*, 2007)[5]
Sa5Y-mCherry	Sa5Y with mTn7-*mCherry*; $Gent^R$	MB_6058	This study
Sa5YΔhlyA-mCherry	Sa5Y with *aph* insertion in *hlyA* (VCA0219) and mTn7-*mCherry*; $Gent^R$, Kan^R	MB_6108	This study
Sa5YΔhapA-mCherry	Sa5Y with *cat* insertion in *hapA* (VCA0865) and mTn7-*mCherry*; $Gent^R$, Cm^R	MB_6109	This study
Sa5YΔhapAΔhlyA-mCherry	Sa5Y with *cat* insertion in *hapA*, *aph* insertion in *hlyA* and mTn7-*mCherry*; $Gent^R$, Cm^R, Kan^R	MB_6130	This study
Sa5YΔlec-mCherry	Sa5Y with *aph* insertion in *lec* (VCA0218 or *tlh*) and mTn7-*mCherry*; $Gent^R$, Kan^R	MB_6110	This study
Sa5YΔlecΔhlyA-mCherry	Sa5Y with *cat* insertion in *lec*, *aph* insertion in *hlyA* and mTn7-*mCherry*; $Gent^R$, Cm^R, Kan^R	MB_6143	This study
A1552ΔrtxA-GFP	A1552 deleted for *rtxA* (VC1451, TransFLP) with mTn7-*gfp*; RifR, Cm^R, $Gent^R$	MB_6120	This study
A1552ΔvipA	A1552 deleted for vipA (TransFLP); Rif^R	MB_3042	(Borgeaud *et al.*, 2015)[3]
A1552ΔvipA-GFP	A1552 deleted for *vipA* (VCA0107, TransFLP) with mTn7-*gfp*; Rif^R, Cm^R, $Gent^R$	MB_6059	This study
A1552ΔvipAΔhlyA-GFP	A1552 deleted for *vipA* and *hlyA* with mTn7-*gfp*; Rif^R, Cm^R, $Gent^R$	MB_6113	This study
ATCC25872	*V. cholerae* non-O1 strain (O37); isolated in 1965, Czechoslovakia	MB_276	(Aldová *et al.*, 1968) [6]

ATCC25872-GFP	ATCC25872 with mTn7-*gfp*; Cm^R, $Gent^R$	MB_5777	This study
ATCC25872ΔvipA	ATCC25872 deleted for *vipA* (TransFLP); Rif^R	MB_5065	This study
ATCC25872ΔvipA-GFP	ATCC25872 deleted for *vipA* (VCA0107, TransFLP) with mTn7-*gfp*; Cm^R, $Gent^R$	MB_5778	This study
ATCC25872ΔhlyA-GFP	ATCC25872 deleted for *hlyA* (VCA0219, TransFLP) with mTn7-*gfp*; Cm^R, $Gent^R$	MB_6062	This study
ATCC25872ΔvipAΔhlyA-GFP	ATCC25872 deleted for *vipA* and *hlyA* with mTn7-*gfp*; Cm^R, $Gent^R$	MB_6064	This study
A1552-vipA-sfGFP	A1552 carrying *vipA-sfgfp* translational fusion (TransFLP); RifR	MB_3036	(Borgeaud *et al.*, 2015)[3]
ATCC25872-vipA-sfGFP	ATCC25872 carrying *vipA-sfgfp* translational fusion (TransFLP)	MB_3038	(Borgeaud *et al.*, 2015)[3]
E. coli			
SM10λpir	thi-1 thr leu tonA lacY supE recA::RP4-2-Tc::Mu, Kmr (λpir); Kan^R	MB_647	(Simon *et al.*, 1983)[7]
S17-1λpir	Tpr Smr recA thi pro hsdR2M1 RP4:2-Tc:Mu:Kmr Tn7 (λpir); Str^R	MB_648	(Simon *et al.*, 1983)[7]
TOP10	F- mcrA Δ(mrr-hsdRMS-mcrBC) φ80lacZΔM15 ΔlacX74 nupG recA1 araΔ139 Δ(ara-leu)7697 galE15 galK16 rpsL(Str^R) endA1λ^-	MB_741	Invitrogen
**Plasmids**			
pUX-BF-13	oriR6K, helper plasmid with Tn7 transposition function; Amp^R	MB_457	(Bao *et al.*, 1991)[8]
pGP704::Tn7-GFP	pGP704 with mini-Tn7 harboring a constitutively expressed *gfp* cassette; AmpR	MB_458	Schoolnik lab collection; (Blokesch, 2012a)[2]
pGP704-mTn7-dsRed	pGP704 with mini-Tn7 carrying *dsRed.T3[DNT]*; Amp^R	MB_1525	(Borgeaud *et al.*, 2015)[3]
pGP704-TnmCherry	pGP704 with mini-Tn7 carrying *mCherry;* Amp^R	MB_5512	This study
pGP704-*hapR*^{Rep}	*hapR* gene and flanking regions on plasmid pGP704-Sac28 to repair frameshift mutation in diverse strains; Amp^R	MB_5648	This study
pBR-flp	FLP^+, λ $cI857^+$, λ p_R from pCP20 integrated into *Eco*RV site of pBR322	MB_1203	(De Souza Silva and Blokesch 2010)[9]
pBR-FRT-Kan-FRT2	pBR322 derivative containing improved FRT-aph-FRT cassette, used as template for TransFLP; Amp^R, Kan^R	MB_3782	(Metzger *et al.*, 2016)[4]
pFRT-aph-pheS*	pBR322 derivative containing improved FRT-aph-FRT cassette as well as site-directly mutated *pheS*[A294G/T251A], used as template for Trans2 method; Amp^R, Kan^R	MB_4591	This study

* VC locus tag numbers according to Heidelberg *et al.* (2000)[10].
# TransFLP method according to De Souza Silva and Blokesch (2010)[9] and Blokesch (2012b)[11]; for Trans2 method, see main manuscript.

Supplementary Table 2. **Quantification of bacterial hemolysin-triggered aberrant amoebal morphologies.**

	Mix (dsRed- & GFP-tagged bacteria)		dsRed-tagged bacteria only		GFP-tagged bacteria only	
**Amoebal morphology***	**aberrant**	**normal**	**aberrant**	**normal**	**aberrant**	**Normal**
WT-dsRed + WT-GFP	4.1 (±1.0)	95.9 (±1.0)	4.8 (±8.2)	95.2 (±8.2)	0.0 (±0.0)	100 (±0.0)
WT-dsRed + ΔhapA-GFP	8.8 (±4.5)	91.2 (±4.5)	0.0 (±0.0)	100 (±0.0)	94.4 (±9.6)	5.6 (±9.6)
WT-dsRed + hlyA↑-GFP	96.3 (±3.2)	3.7 (±3.2)	13.9 (±12.7)	86.1 (±12.7)	100 (±0.0)	0.0 (n.a.) [#]

*Percentage of observed morphotypes. Morphotypes (normal or aberrant) were scored under the indicated co-culturing conditions. Both bacterial strains were mixed at a 1:1 ratio prior to infection. Given are percentages of infected amoebae (± standard deviation). Values are based on three independent biological replicates with a number of counted amoebae per experiment equal to 2000 (n=6000 in total). [#]Single event observed out of 6000 amoebae counted; n.a. = not applicable.

Supplementary Table 3. Oligonucleotides used in this study.

Primer name	Primer sequence (5' to 3')	Comments	Internal code
Insert_Tn_fwd	GTACTGAGATTGGTGTGGCTTCAACC	To amplify mTn7-*mCherry* from gDNA of strain A1552 Tn-mCherry to then transform strain SA5Y with resulting PCR fragment.	Box 25/48
Insert_Tn_bwd	GCCGCATAACCTCTTGTCGTTTCCG		Box 25/49
Insert_Tn-chk_up	GAAAGGCTATATGGCGGCGATGACG	To check insertion of mTn7-*mCherry* in strain SA5Y.	Box 25/47
Insert_Tn-chk_dwn	TCTTCGATGCCGTTTGGCATGATCG		Box 25/50
hapA_1	TCGCGGGTGGTTATAAGTTCTAACG	Used to construct PCR fragments A (#1 and #2), B (#3 and #4), C (#5 and #6) and D (#1 and #6) to insert *cat* gene into *hapA* gene (VCA0865) of strains A1552, N16961-hapR repaired, C6706-original, and SA5Y (TransFLP method).	Box 50/34
hapA_2	AGCTCCAGCCTACGCACGCACTTTCACCTTACCATTCGG		Box 50/35
hapA_3	TGAAAGTGCGTGCGTAGGCTGGAGCTGCTTCGAAGTTCC		Box 50/36
hapA_4	TGCGATATCCGGATGAATATCCTCCTTAGTTCCTATTCC		Box 50/37
hapA_5	AGGAGGATATTCATCCGGATATCGCAGGGGAAGCGGCA G		Box 50/38
hapA_6	TATGGACGACAATCCCAAGAAGAGG		Box 50/39
hapA_chk-up	TCCTCAACGCTTCTGTGTGGTATGC	To check insertion and flipping of *cat* gene in *hapA* (VCA0865).	Box 50/40
hapA_chk-dw	TAGGTGGCAAGGAAGTTAGTCCAAGCG		Box 50/41
LacZ FRT1	ATGCGCAACTTCTCCGATATTCTTCTTAGCC	Construction of complemented strains for *hapA*. Amplifications: PCR A (LacZ FRT1/hapA-compl_2), PCR B (hapA-compl_3/hapA-compl_4), PCR C (hapA-compl_5/lacZ_FRT4). PCR product was used to transform *V. cholerae* strains.	Box 8/74
hapA-compl_2	AACTATTTGAGTTTTCAATCGCGAGTATCGGCTTGCGG		Box 69/15
hapA-compl_3	ACTCGCGATTGAAAACTCAAATAGTTGATACATCCTAG		Box 69/16
hapA-compl_4	GTGTGATTTTAGGCTCCATTCAGAGCCTGAGACGTACG		Box 69/17
hapA-compl_5	GCTCTGAATGGAGCCTAAAATCACACACGCTCCGCTGCC		Box 69/18
lacZ_FRT4	GAGATACCACTTATCGCCCGCCACCAACTCG		Box 8/73
lacZ-before-fw	ATTGGTGAGTGGTTCACAGAATCGG	To check for complementing constructs within *lacZ* gene (e.g., *hapA, lec, hlyA*).	Box 53/23
lacZ-end-rev	CGTTACCCAATCCAACTGATGGCAG		Box 53/24
hlyA_1	GTCTTTAGAGGCTAAAATCTGTGATCCGC	Used to construct PCR fragments A (#1 and #2), B (#3 and #4), C (#5 and #6) and D (#1 and #6) to insert *aph* gene into *hlyA* gene (VCA0219) of strains A1552, N16961-hapR repaired, C6706-original, SA5Y and ATCC25872 (TransFLP method).	Box 50/26
hlyA_2	AGCTCCAGCCTACGCTAATATTGTGAATATCGCGATTGC		Box 50/27
hlyA_3	TCACAATATTAGCGTAGGCTGGAGCTGCTTCGAAGTTCC		Box 50/28
hlyA_4	TGGGTGATCCGGATGAATATCCTCCTTAGTTCCTATTCC		Box 50/29
hlyA_5	AGGAGGATATTCATCCGGATCACCCAGTATTCACGGGTG		Box 50/30
hlyA_6	CCATATTGGAAATCACCAGAGGTTGC		Box 50/31
hlyA_chk_up	GCGTATTGAAATCTTTAGAGTTAAAATGG	To check insertion and flipping of *aph* gene in *hlyA* (VCA0219).	Box 50/32
hlyA_chk_down	ACGGTCTTCATGAGCATTGG		Box 50/33
compl-hlyA_2	GTACGCAATCCGGATGAATATCCTCCTTAGTTCCTATTCC	Construction of *hlyA*-complemented strains. Amplifications: PCR A (LacZ FRT1/compl-hlyA_2), PCR B (tlh_comp-3/compl-hlyA_2), PCR C (compl-hlyA_3/compl-hlyA_4), PCR D (compl-hlyA_5/lacZ_FRT4), and PCR E (LacZ FRT1/lacZ_FRT4). PCR product was used to transform *V. cholerae* strains (TransFLP method).	Box 63/10
compl-hlyA_3	GGAGGATATTCATCCGGATTGCGTACTTTTACCCTAATG TG		Box 63/11
compl-hlyA_4	GTGTGATTTTAGGGCGTATTGAAATCTTTAGAGTTAAAA TGG		Box 63/12
compl-hlyA_5	GATTTCAATACGCCCTAAAATCACACACGCTCCGCTGCC		Box 63/13
LacZ-constHlyA_2	AAAGTACGCAATCTTCAATCGCGAGTATCGGCTTGCGG	Construction of strain with constitutive *hlyA* expression (hlyA↑; strain A1552-hlyA↑). Amplifications of fragment *hlyA* preceded by strong PA1/04/03 promoter; PCR A (LacZ FRT1/LacZ-	Box 63/14
LacZ-constHlyA_3	ACTCGCGATTGAAGATTGCGTACTTTTACCCTAATGTG		Box 63/15

LacZ-constHlyA_4	GGAGAAATTAAGCATGCCAAAACTCAATCGTTGCGCAATCG	constHlyA_2), PCR B (LacZ-constHlyA_3/ LacZ-constHlyA_4), PCR C (LacZ-constHlyA_5/ lacZ_FRT4), and PCR D (LacZ FRT1/lacZ_FRT4). PCR product was used to transform *V. cholerae* strains (Trans2 method).	Box 63/16
LacZ-constHlyA_5	TGAGTTTTGGCATGCTTAATTTCTCCTCTTTAATTCTAGATG		Box 63/17
hlyA-end	GTCTGACGATTGGTTGGTGAAAGG	Primers used to sequence the complementing and constitutively expressing *hlyA* construct within *lacZ*.	Box 63/18
hlyA-SEQ1	AGGCATTAGACGCATTGCAACCCTG		Box 63/19
hlyA-SEQ2	AAACTTACCGCATCGTTGCTGCTC		Box 63/20
hlyA-SEQ3	TGAAGGTCAAGCAGAGATGCAAGC		Box 63/21
hlyA-SEQ4	GGTATTTGTTTCGCTGTTTGCCGG		Box 63/22
VCA0218_1	GCC TAC CTG CAA CTC CAA GGA GTT TTG G	Used to construct PCR fragments A (#1 and #2), B (#3 and #4), C (#5 and #6) and D (#1 and #6) to insert *aph* or *cat* gene in *lec* gene (VCA0218 or *tlh*) of strains A1552, N16961-hapR repaired, C6706-original and SA5Y (TransFLP method).	Box 52/68
VCA0218_2	AGCTCCAGCCTACGCTAAGCTAGCTAAGCCAGCGATTAG		Box 52/69
VCA0218_3	TAGCTAGCTTAGCGTAGGCTGGAGCTGCTTCGAAGTTCC		Box 52/70
VCA0218_4	CTGATAGTTCGGATGAATATCCTCCTTAGTTCCTATTCC		Box 52/71
VCA0218_5	AGGAGGATATTCATCCGAACTATCAGCCAGAAAACAGCT		Box 52/72
VCA0218_6	GGAAATTGTGCCATTTCGGTGGCC		Box 52/73
VCA0218_chk_UP	GCATATCCCCTTCTTACTCGAAGCGG	To check insertion and flipping of *aph* or *cat* genes in *lec* (VCA0218 or *tlh*).	Box 52/74
VCA0218_chk_DOWN	GTAACTCTGAACCGGTAAAAGCAGG		Box 52/75
tlh_comp-2	AGCTCCAGCCTACGCTTCAATCGCGAGTATCGGCTTGCG	Construction of complemented or merodiploid strain of *lec* (VCA0218 or *tlh*) in *V. cholerae*. Amplifications: PCR 1 (LacZ FRT1/tlh_comp-2), PCR 2 (tlh_comp-3/tlh_comp-4), PCR 3 (tlh_comp-5/tlh_comp-6), PCR 4 (tlh_comp-7/lacZ_FRT4) and PCR 5 (LacZ FRT1/lacZ_FRT4). PCR product was used to transform *V. cholerae* starins. Resistance cassette (aph) was flipped out afterwards from the transformants (TransFLP method).	Box 54/41
tlh_comp-3	TCGCGATTGAAGCGTAGGCTGGAGCTGCTTCGAAGTTCC		Box 54/42
tlh_comp-4	TCAGTCTCACGGATGAATATCCTCCTTAGTTCCTATTCC		Box 54/43
tlh_comp-5	AGGAGGATATTCATCCGTGAGACTGAGGATGGATTTACG		Box 54/44
tlh_comp-6	GTGTGATTTTAGGCTCTAAAGATTTCAATACGCTTACAC		Box 54/45
tlh_comp-7	GAAATCTTTAGAGCCTAAAATCACACACGCTCCGCTGCC		Box 54/46
VCA0107-KO_1	CAGCGATCGCTTGAGTCATGTCTACC	Used to construct PCR fragments A (#1 and #2), B (#3 and #4), C (#5 and #6) and D (#1 and #6) to insert *aph* gene in *vipA* gene (VCA0107) of strains A1552 and ATCC25872. Followed by flipping and curing, therefore rendering strains deleted for *vipA* (TransFLP method).	Box 39/21
VCA0107-KO_2	AGCTCCAGCCTACGCGATATTAATCCGCTCTTTGGGAGC		Box 39/22
VCA0107-KO_3	GGATTAATATCGCGTAGGCTGGAGCTGCTTCGAAGTTCC		Box 39/23
VCA0107-KO_4	AATATACTTCATATGAATATCCTCCTTAGTTCCTATTCC		Box 39/24
VCA0107-KO_5	AGGAGGATATTCATATGAAGTATATTCCGGCGACGGGG		Box 39/25
VCA0107-KO_6	CAAGAAGGCTGCCTTGAGCAAGCTGTG		Box 39/26
VCA0107-KO_chk-up	GATCTCACTTCTTTGGTGGCGATGGG	To check insertion and flipping of *aph* casette in *vipA* gene (VCA0107).	Box 39/27
VCA0107-KO_chk-dw	CAGGCTCAGCAGAGTGTTGTGAACCC		Box 39/28
KAN-FRT-START-BACK	CTGGCTTTCTACGTGTTCCGCTTCC	To check insertion of *aph* (outwards facing primer).	Box 8/80
cat-end-out	ATTACAACAGTACTGCGATGAGTGGC	To check insertion of *cat* (outwards facing primer).	Box 40/55
VC0487-end-out	GCATGTTAGCGAGATTGTGGCTCCG	To check transposon insertion at correct chromosomal locus after triparental mating (to insert mTn7-*gfp* and mTn7-*mCherry* into different strains).	Box 4/47
NotI-90-outwards	GCTTTAGCCATAACAAAAGTCCAG		Box 4/48

NotI-Gent	ATCACCGGATCCCGACGGGCCCGG	PCR amplification of *mCherry* preceded by promoter PA1/04/03 using a synthetic DNA construct as template (synthesized by IDA; Integrated DNA Technologies) to construct mTn7-*mCherry*.	Box 4/49
SYN-Not-rev	ACTAGGGCTAATTCGGGCCAGTTGC		Box 73/54
TnR7R_before	AAAGGATCCTCTAGAGGACCAGCC	Primers used to screen for genomic insertion of mTn7-mCherry after triparental mating.	Box 33/19
pBADseq-down	GATGATGGTCGACGGCGCTATTCAGATCC		Box 5/19
LacZ-K-phe-4	GATTTTAGGCGGATGAATATCCTCCTTAGTTCCTATTCC	Construction of strain ΔlacZ::pheS* (with fragment ΔlacZ::FRT-Kan-pheS*-FRT). Amplifications: PCR A (LacZ FRT1/tlh_comp-2), PCR B (tlh_comp-3/LacZ-K-phe-4), PCR C (LacZ-K-phe-5/lacZ_FRT4), and PCR D (LacZ FRT1/lacZ_FRT4). PCR product was used to transform *V. cholerae* strains.	Box 60/77
LacZ-K-phe-5	AGGAGGATATTCATCCGCCTAAAATCACACACGCTCCGC		Box 60/78
flaA FRT-1	CGGATGCCTCGAATACGGCCAAACTGCATC	Used to construct PCR fragments A (flaA FRT-1/flaA FRT-2), B (flaA FRT-5/flaA FRT-6), C (flaA FRT-3/flaA FRT-4) and D (flaA FRT-1/flaA FRT-4) to insert *aph* gene in *flaA* gene of strain A1552. Followed by flipping and curing, therefore rendering strain deleted for *flaA* (TransFLP method).	Box 15/57
flaA FRT-2	GCTCCAGCCTACGCTTACACGTTGGTATTTACGTTAA		Box 15/58
flaA FRT-3	GGAGGATATTCATATGGCAATCTCGTTATTGCAGTAG		Box 15/59
flaA FRT-4	GTATCCTGAAGATAGACGTTCCATTGACGATTGTTG		Box 15/60
flaA FRT-5	ACCAACGTGTAAGCGTAGGCTGGAGCTGCTTCGAA		Box 15/61
flaA FRT-6	ACGAGATTGCCATATGAATATCCTCCTTAGTTCCTATTCC		Box 15/62
flaA chk-up	CTGACGCCTCGCACCACGTTTAGTG	To check insertion and flipping of *aph* cassette in *flaA* gene.	Box 15/63
flaA chk-down	CCTAAACCTCGGCTTTGCACGTTCAAG		Box 15/64
pomB_1	TGATGCTCCCGAAGATCTGATTGC	Used to construct PCR fragments A (#1 and #2), B (#3 and #4), C (#5 and #6) and D (#1 and #6) to insert *aph* gene in *pomB* of strain A1552. Followed by flipping and curing, therefore rendering strain deleted for *pomB* (TransFLP method).	Box 60/50
pomB_2	AGCTCCAGCCTACGCTTGTTCGTCATCCATAGTCGGCTC		Box 60/51
pomB_3	ATGACGAACAAGCGTAGGCTGGAGCTGCTTCGAAGTTCC		Box 60/52
pomB_4	TTGGTTATCCGGATGAATATCCTCCTTAGTTCCTATTCC		Box 60/53
pomB_5	AGGAGGATATTCATCCGGATAACCAAAACACTCAATCTG		Box 60/54
pomB_6	TGAAGGAACCTCGTCACTGTAGACTG		Box 60/55
pomB_chk-up	TGGTATCAGTTCTTATCGTGGTCGGC	To check insertion and flipping of *aph* cassette in *pomB* gene.	Box 60/56
pomB_chk-dw	TGAGTCCCCATGAGCATAGATAGA C		Box 60/57
rtxA_1	TTGTTGCTGCTGGATTTCTGCCAGC	Used to construct PCR fragments A (#1 and #2), B (#3 and #4), C (#5 and #6) and D (#1 and #6) to insert *aph* gene in *rtxA* (VC1451) gene of strain A1552. Followed by flipping and curing, therefore rendering strain deleted for *rtxA* (TransFLP method).	Box 51/30
rtxA_2	AGCTCCAGCCTACGCAAATGGTTTTCCCATAAGCCAAAC		Box 51/31
rtxA_3	GAAAACCATTTGCGTAGGCTGGAGCTGCTTCGAAGTTCC		Box 51/32
rtxA_4	ACCTTCTTTCGGATGAATATCCTCCTTAGTTCCTATTCC		Box 51/33
rtxA_5	AGGAGGATATTCATCCGAAAGAAGGTGATCACACGGCC		Box 51/34
rtxA_6	CCGATTTGGGTATGGGTGTTGTAGC		Box 51/35
rtxA_chk-up	GCAAGTTGTTCATATGCCTGCATCC	To check insertion and flipping of *aph* cassette in *rtxA* gene.	Box 51/36
rtxA_chk-dw	CCATTTTGGTCAGTACGTTTGCACC		Box 51/37

hapR-repair-NcoI	GGACCCATGGAGCTAGAGGCGGTCATAAAATCGAC	Used to amplify *hapR* gene from strain A1552 and clone into suicide plasmid, resulting in plasmid pGP704-$hapR^{Rep}$; used to repair *hapR* frameshift mutation in strain N16961.	Box 75/60
hapR-repair-SacI	CAACGAGCTCTTGCCCGTGAACTCACCAAAACC		Box 75/61
hapR-repair-PCRchk	TTTCAGAATGCGCACATCACGTGAGC	Used to check for *hapR* exchange and repair.	Box 75/62
VC0582-end-fwd	AAATGGGGCTTGGAGAATTTAGGCG		Box 75/63
FKpheSF-1-P	TAAGCGTAGGCTGGAGCTGCTTCGAAGTTCC	Used to construct PCR fragments A (FKpheSF-1-P/FKpheSF-2), B (FKpheSF-3/FKpheSF-4), C (FKpheSF-5/FKpheSF-6-P) and D (FKpheSF-1-P/FKpheSF-6-P) to construct plasmid pBR-FKpheSF (precursor of plasmid pFRT-aph-pheS*).	Box 58/48
FKpheSF-2	TCTGCTAATCAGCCGTCGCTTGGTCGGTCATTTCGAAC		Box 58/49
FKpheSF-3	GACCAAGCGACGGCTGATTAGCAGACGAGGAAACGATG		Box 58/50
FKpheSF-4	GGCAGGTTGGGTCTTGGGAACCCTGAATTATTTGAACTG		Box 58/51
FKpheSF-5	CAGGGTTCCCAAGACCCAACCTGCCATCACGAGATTTCG		Box 58/52
FKpheSF-6-P	CGGATGAATATCCTCCTTAGTTCCTATTCCGAAGTTCC		Box 58/53
pheS[A294G]-inv1	ATACCAAAACCGAATCCGGAGTATTTCTCAGGGTCGATG	Used to mutate plasmid pBR-FKpheSF by inverse PCR resulting in plasmid pBR-FKpheSF[A294G] (precursor of plasmid pFRT-aph-pheS*).	Box 58/60
pheS[A294G]-inv2	AATACTCCGGATTCGGTTTTGGTATGGGCGTTGAGCG		Box 58/61
pheS[T251A]-inv1	CACTTCAGCTGAAGGCTCAGCGAACGGGAAGTAAGACG G	Used to mutate plasmid pBR-FKpheSF[A294G] by inverse PCR resulting in plasmid pFRT-aph-pheS*.	Box 58/62
pheS[T251A]-inv2	CGTTCGCTGAGCCTTCAGCTGAAGTGGATGTCAAAGGC		Box 58/63
FKpheSF-chk-up	CCTCTTGCGGGATTAAGCGTAGGCTG	Primers used to screen for right plasmid and to sequence plasmids pBR-FKpheSF, pBR-FKpheSF[A294G], and pFRT-aph-pheS*	Box 58/54
FKpheSF-chk-dw	CCTCGCGTCGCGGATGAATATCCTC		Box 58/55
Before-EcoRV-SEQ	AACAATGCGCTCATCGTCATCCTCG		Box 58/56
aph-mid-SEQ	CTGTGCTCGACGTTGTCACTGAAGC		Box 58/57
pheS-mid-SEQ	TGACGGTTCAACTGCAAAGCCTAGG		Box 58/58
pheS-A294-up	CGGCAAATGGCTAGAAGTGCTAGG		Box 58/59
pheS-T251-up	GTGTTTACCGTAACGACTACGACC		Box 58/64
pheS-T251-dw	CGCAGATCGTTTTCGAAGAATGCACG		Box 58/65
pBR-TET_MCSafter	ATCATGCGCACCCGTGGCCAGGACCC		Box 7/1
gyrA-157-fwd	AATGTGCTGGGCAACGACTG	Primers used for qRT-PCR (*gyrA* transcript level).	Box 19/18
gyrA_332_bwd	GAGCCAAAGTTACCTTGGCC		Box 19/19
hapR-230-fwd	CCAACTTCTTGACCGATCAC	Primers used for qRT-PCR (*hapR* transcript level).	Box 19/24
hapR-399-bwd	GGTGGAAACAAACAGTGGCC		Box 19/25
hapA_175_fwd	ACGGTACAGTTGCCGAATGG	Primers used for qRT-PCR (*hapA* transcript level).	Box 19/32
hapA_358_bwd	GCTGGCTTTCAATGTCAGGG		Box 19/33
qRT_hlyA_fwd	ATGAACCAAGTGGTGAAGCG	Primers used for qRT-PCR (*hlyA* transcript level).	Box 44/27
qRT_hlyA_rev	CAGCATCACTGATCTGACTG		Box 44/28

Supplementary References

1. Yildiz, F. H. & Schoolnik, G. K. Role of *rpoS* in stress survival and virulence of *Vibrio cholerae*. *J. Bacteriol.* **180**, 773-784, (1998).
2. Blokesch, M. Chitin colonization, chitin degradation and chitin-induced natural competence of *Vibrio cholerae* are subject to catabolite repression. *Environ. Microbiol.* **14**, 1898-1912, (2012).
3. Borgeaud, S., Metzger, L. C., Scrignari, T. & Blokesch, M. The type VI secretion system of *Vibrio cholerae* fosters horizontal gene transfer. *Science* **347**, 63-67, (2015).
4. Metzger, L. C. *et al.* Independent Regulation of Type VI Secretion in *Vibrio cholerae* by TfoX and TfoY. *Cell Rep.* **15**, 951-958, (2016).
5. Keymer, D. P., Miller, M. C., Schoolnik, G. K. & Boehm, A. B. Genomic and phenotypic diversity of coastal *Vibrio cholerae* strains is linked to environmental factors. *Appl. Environ. Microbiol.* **73**, 3705-3714, (2007).
6. Aldova, E., Laznickova, K., Stepankova, E. & Lietava, J. Isolation of nonagglutinable vibrios from an enteritis outbreak in Czechoslovakia. *J. Infect. Dis* **118**, 25-31, (1968).
7. Simon, R., Priefer, U. & Pühler, A. A broad host range mobilization system for *in vivo* genetic engineering: transposon mutagenesis in Gram negative bacteria. *Nat. Biotechnol.* **1**, 784-791, (1983).
8. Bao, Y., Lies, D. P., Fu, H. & Roberts, G. P. An improved Tn*7*-based system for the single-copy insertion of cloned genes into chromosomes of Gram-negative bacteria. *Gene* **109**, 167-168, (1991).
9. De Souza Silva, O. & Blokesch, M. Genetic manipulation of *Vibrio cholerae* by combining natural transformation with FLP recombination. *Plasmid* **64**, 186-195, (2010).
10. Heidelberg, J. F. *et al.* DNA sequence of both chromosomes of the cholera pathogen *Vibrio cholerae*. *Nature* **406**, 477-483, (2000).
11. Blokesch, M. TransFLP–a method to genetically modify *V. cholerae* based on natural transformation and FLP-recombination. *J. Vis. Exp.* **68**, e3761, (2012).
